# Involuntary and voluntary recall of musical memories: A comparison of temporal accuracy and emotional responses

**DOI:** 10.3758/s13421-018-0792-x

**Published:** 2018-01-29

**Authors:** Kelly Jakubowski, Zaariyah Bashir, Nicolas Farrugia, Lauren Stewart

**Affiliations:** 1 Department of Music, Durham University, Palace Green, Durham, DH1 3RL UK; 2 Department of Psychology, Goldsmiths, University of London, London, UK; 3 IMT Atlantique, Brest, France; 4 Center for Music in the Brain, Department of Clinical Medicine, Aarhus University and The Royal Academy of Music Aarhus, Aalborg, Denmark

**Keywords:** Involuntary memory, Musical imagery, Earworms, Tempo

## Abstract

Comparisons between involuntarily and voluntarily retrieved autobiographical memories have revealed similarities in encoding and maintenance, with differences in terms of specificity and emotional responses. Our study extended this research area into the domain of musical memory, which afforded a unique opportunity to compare the *same* memory as accessed both involuntarily and voluntarily. Specifically, we compared instances of involuntary musical imagery (INMI, or “earworms”)—the spontaneous mental recall and repetition of a tune—to deliberate recall of the same tune as voluntary musical imagery (VMI) in terms of recall accuracy and emotional responses. Twenty participants completed two 3-day tasks. In an INMI task, participants recorded information about INMI episodes as they occurred; in a VMI task, participants were prompted via text message to deliberately imagine each tune they had previously experienced as INMI. In both tasks, tempi of the imagined tunes were recorded by tapping to the musical beat while wearing an accelerometer and additional information (e.g., tune name, emotion ratings) was logged in a diary. Overall, INMI and VMI tempo measurements for the same tune were strongly correlated. Tempo recall for tunes that have definitive, recorded versions was relatively accurate, and tunes that were retrieved deliberately (VMI) were not recalled more accurately in terms of tempo than spontaneous and involuntary instances of imagined music (INMI). Some evidence that INMI elicited stronger emotional responses than VMI was also revealed. These results demonstrate several parallels to previous literature on involuntary memories and add new insights on the phenomenology of INMI.

## Involuntary and voluntary memories

Although most classic memory studies have focused on understanding memories that are retrieved via strategic, deliberate search processes, recent research has highlighted the importance of involuntary memory retrieval as a frequent and prevalent experience across the lifespan (Berntsen, 1996, 2010; Rubin & Berntsen, 2009). Involuntary memory processes appear to be more than mere attentional lapses or failures in executive control; rather, such memories may serve functions in their own right, including maintaining continuity of the life narrative and aiding problem solving via fast, effortless access to memories with distinctive cue overlaps with the current situation (Rasmussen & Berntsen, 2009).

A body of literature has now accumulated that compares involuntarily versus deliberately/voluntarily retrieved memories, with the majority of such research focused on comparing involuntary autobiographical memories (IAMs) to voluntary autobiographical memories (VAMs; e.g., Berntsen, 1998; Berntsen & Hall, 2004; Johannessen & Berntsen, 2010; Mace, 2006; Rubin & Berntsen, 2009; Schlagman & Kvavilashvili, 2008). Overall, this literature suggests that IAMs are “a basic mode of remembering that operates on the same episodic memory system as voluntary (strategic) remembering and thus follows the same rules of encoding and maintenance” (Berntsen, 2010, p. 138); this account is in contrast to earlier theories based on clinical evidence in which two separate memory systems were proposed (e.g., Brewin, Dalgleish, & Joseph, 1996). Neuroimaging evidence is consistent with this unitary system view, as differences in brain activation patterns have been found to be predominantly associated with initial retrieval mode (involuntary vs. voluntary), whereas similar activation patterns are associated with retrieval success following both involuntary and voluntary retrieval of episodic memories (Hall et al., 2014).

Despite these similarities in encoding and maintenance processes, several differences between IAMs and VAMs have been revealed in both laboratory and naturalistic contexts (e.g., diary studies). These include that: (1) IAMs tend to be of specific episodes, while generic recall of summarized events is more prevalent in VAMs (Berntsen, 1998; Berntsen & Hall, 2004; Schlagman & Kvavilashvili, 2008), (2) IAMs tend to be accompanied by a greater degree of reliving and stronger emotional responses than VAMs (Berntsen & Hall, 2004; Rubin, Boals, & Berntsen, 2008), and (3) IAMs tend to be retrieved significantly faster than VAMs (Schlagman & Kvavilashvili, 2008). One question that remains unanswered here is whether such differences also emerge when comparing the *same* memory as accessed via both retrieval modes, as the vast majority of previous studies have compared *different* sets of memories that were recalled involuntarily versus voluntarily, in either between- or within-subjects designs.^1^ In the present paper, a novel paradigm will be introduced that was developed for studying the same memory in a within-subjects design, in which only the retrieval mode varied between the two tasks.

## Involuntary and voluntary musical memories

The present work adds a new dimension to research on involuntarily versus voluntarily retrieved memories by extending this comparison to the domain of musical memory. Several commonalities between musical memories and autobiographical memories have already been revealed in previous research. For instance, older adults remember more details and respond more emotionally to music from their adolescence than other music (Schulkind, Hennis, & Rubin, 1999), which is similar to the “reminiscence bump” that is observed for autobiographical memories (e.g., Rubin & Schulkind, 1997). Long-term recall of both musical and autobiographical memories also appears to be facilitated by emotional intensity of the memory (Eschrich, Münte, & Alltenmüller, 2008; Samson, Dellacherie, & Platel, 2009; Thompson, 1985; Walker, Vogl, & Thompson, 1997). Involuntary memories for music—which have sometimes been classified as involuntary *semantic* memories (Kvavilashvili & Anthony, 2012; Kvavilashvili & Mandler, 2004)—show a similar prevalence in everyday life to IAMs (Berntsen, 2010; Liikkanen, 2012), but have not been compared in detail to their voluntarily retrieved counterparts. Thus, our study aimed to use similar methods to those employed in autobiographical memory research to test how retrieval mode affects aspects of musical memory recall.

The type of involuntary musical memory that we focused on in this work was involuntary musical imagery (INMI, or “earworms”)—the spontaneous recall and repetition of a piece of music within the mind. Previous research on INMI has focused on such topics as identifying common triggers of INMI episodes and the contexts in which INMI is most likely to occur (Floridou, Williamson, & Stewart, 2017; Hyman et al., 2013; Williamson et al., 2012), individual differences in personality, musical background, and cognitive styles that predict INMI frequency and reactions to INMI (Beaman & Williams, 2013; Beaty et al., 2013; Liikkanen, 2012; Müllensiefen et al., 2014), strategies for alleviating unwanted INMI (Beaman & Williams, 2010; Williamson, Liikkanen, Jakubowski, & Stewart, 2014), and commonalities between musical features of frequently-reported INMI tunes (Jakubowski, Finkel, Stewart, & Müllensiefen, 2017). Some parallels to IAMs have also been revealed. For instance, IAMs and INMI both occur more frequently during periods of diffused attention, when a person is not focused on a particular task (Berntsen, 1998; Floridou et al., 2017; Williamson et al., 2012). In addition, IAMs and INMI both appear to be relatively harmless, everyday manifestations of thought processes that can also emerge in more extreme, clinical versions (intrusive memories and musical obsessions, respectively; Brewin, 1998; Taylor et al., 2014). However, studies comparing aspects of the INMI experience to voluntarily retrieved musical memories have been infrequent.

One previous study examined whether participants who reported experiencing more frequent INMI performed more accurately on a task requiring generation of voluntary musical imagery (VMI) (Weir, Williamson, & Müllensiefen, 2015). If this hypothesis were supported it could suggest that INMI and VMI rely on similar mental rehearsal mechanisms, such that regular involuntary activation and repetitive rehearsal of musical memories could influence aspects of voluntarily imagined music (specifically, Weir et al. examined acuity of pitch and timing information within VMI). However, no significant relationship between INMI frequency and VMI task performance was found. This could suggest that INMI and VMI rely on different rehearsal mechanisms. Alternatively, it could be that the retrospective, self-report INMI frequency measure used by Weir et al. (2015) was not reliable or fine-grained enough to detect differences between participants, or that the relatively specific abilities measured by the VMI task are not developed by frequent INMI experiences. Beaty et al. (2013) examined both INMI and VMI experiences during everyday life using the Experience Sampling Method, although the main focus of this study was not on comparing the two types of imagery and most analyses focused on musical imagery experiences as a whole, without differentiating between retrieval modes. This work revealed that only a small proportion of imagery episodes were rated as being imagined “on purpose,” suggesting that INMI may be more frequent than VMI in everyday life. Participants also tended to report more purposeful musical imagery when they were in a happy mood. Thus, it may be that deliberate control over imagined music is a contributor to positive mood, although causation cannot be ascertained from this correlational result.

## The present study

The aim of the present study was to extend the literature on involuntary memory by comparing INMI and VMI, as experienced by the same participant for the same piece of music. As mentioned previously, comparison of the *same* memory as retrieved in an involuntary versus voluntary fashion in a within-subjects design has been rare in involuntary memory research. Music research represents a useful forum for implementing such a design, as participants can easily be instructed to voluntarily imagine the same version and same section of a tune they have previously reported as INMI. In addition, the present study adds a new dimension to this research area by comparing recall *accuracy* between involuntarily and voluntarily retrieved memories. This can be achieved by comparing features of the INMI and VMI experience to features of the original recording for tunes that have a definitive, canonical version.^2^ In the present study, we focused specifically on accuracy of musical tempo recall. In the autobiographical memory literature, objective assessments of accuracy have often not been possible because the experimenter was not present at the point of encoding for the memories of interest (e.g., childhood memories), although one previous study has reported that IAMs obtained similar ratings of confidence in the remembered details to VAMs, as judged by the study participants and acquaintances who were present during the remembered event (Mace, Atkinson, Moeckel, & Torres, 2011). Musical memory paradigms present a more direct opportunity to assess accuracy, by comparing aspects of the recalled music to the original music, which allows for a fine-grained and relatively objective assessment of memory accuracy.

The present study built on work by Jakubowski, Farrugia, Halpern, Sankarpandi, and Stewart (2015), which examined temporal and mood-related aspects of the INMI experience. In this study, INMI tempo was measured during the course of participants’ everyday lives by asking them to tap to the beat of INMI episodes while wearing a wrist-mounted accelerometer. Additional information about the INMI tune and concurrent mood state was recorded in a paper diary. It was found that INMI episodes for canonical tunes were generally recalled close to the original tempo (mean deviation from the original tempo = 10.8 %). This high precision of tempo recall has been corroborated in subsequent work in which INMI episodes were sung into a recording device, with a reported mean deviation from the original tempo of approximately 12 % (McNally-Gagnon, 2016). Comparisons to a separate study in which participants were asked to tap the tempo of pop songs in a VMI task (Jakubowski, Farrugia, & Stewart, 2016) suggest that musical tempo recall within INMI is as, or perhaps even more, accurate than tempo recall within VMI (mean deviation from the original tempo in VMI task = 18.4 %). However, this difference could potentially be attributed to design differences between the studies; for instance, Jakubowski et al. (2016) was a laboratory-based study using experimenter-selected pop songs whereas Jakubowski et al. (2015) was a naturalistic, diary study in which participants recorded all INMI episodes as they occurred. In addition, potentially confounding variables such as familiarity with a tune and recency of hearing it aloud cannot be accounted for when comparing these studies.

Our study extended the paradigm of Jakubowski et al. (2015) by adding an analogous VMI task following the initial INMI task. In this VMI task, participants were prompted via text message to imagine the same section of a tune that had previously been reported as INMI, tap to the beat of the tune while wearing the accelerometer, and record information about the VMI experience in a diary. The primary aims of our study were to test: (1) the accuracy of tempo recall within INMI versus VMI, as compared to the original tempo for songs that have a definitive, recorded version, and (2) emotional responses to the same music experienced as INMI versus VMI. Predictions related to each of these aims, as informed by existing literature, are discussed below.

1. *How accurate is tempo recall for INMI versus VMI?*

The present study is the first to compare recall accuracy between involuntary and voluntary musical memories within the same paradigm and same participants. In the autobiographical memory domain, one study has revealed similar subjective ratings for the accuracy of details when comparing IAMs to VAMs for different autobiographical events (Mace et al., 2011). In the musical memory literature, previous evidence accumulated across three separate studies (Jakubowski et al., 2015, 2016; McNally-Gagnon, 2016) indicates that INMI tempo may potentially be recalled *more* accurately than VMI tempo, although methodological differences between the studies limit the extent to which definitive conclusions can be drawn. If INMI tempo is recalled with similar accuracy to VMI tempo, this would suggest that the findings of Mace et al. (2011) generalize to musical memories as well as to accuracy comparisons for the same memory as recalled via both retrieval modes. If INMI tempo is found to be recalled more accurately than VMI tempo, this could relate to the greater degree of reliving that has previously been attributed to IAMs in comparison to VAMs (Berntsen & Hall, 2004). In musical imagery, heightened reliving could manifest as increased vividness of the mental image, and perhaps a more veridical mental representation. Additionally, the autobiographical memory literature indicates that voluntarily retrieved memories tend to favor more generic, summarized representations of events, whereas IAMs tend more often to be of specific events (Bernsten, 1998; Berntsen & Hall, 2004; Schlagman & Kvavilashvili, 2008). More generic representations of musical memories could result in a decrease in veridicality, thus leading to less accurate tempo recall within VMI in comparison to INMI. The present design will also take account of recent hearing of a piece of music as a potential confounding variable that could influence accuracy of tempo recall in both the INMI and VMI tasks.

2) *How emotional are responses to INMI versus VMI?*

The autobiographical memory literature has revealed that IAMs elicit more immediate and heightened emotional responses in comparison to VAMs (Berntsen & Hall, 2004; Rubin, Boals, & Berntsen, 2008), which may be attributed to the unprepared nature of involuntary memory experiences (Berntsen, 2010). The present study tested whether heightened emotional responses would also be revealed for involuntary musical memories, as compared to the same musical memory accessed via voluntary retrieval. It was also predicted that emotional responses to the imagined music might be mediated by features of the music itself. In particular, Jakubowski et al. (2015) found a significant positive relationship between INMI tempo and concurrent arousal ratings, but no relationship between INMI tempo and positive/negative mood. Thus, we also aimed to replicate this result and test whether a similar relationship might be found between VMI tempo and arousal.

In sum, the present work aimed to add to the growing body of knowledge on the comparison of involuntarily versus voluntarily recalled memories, to test whether previous effects found for autobiographical memories generalize to other types of memories, and to provide new insights on whether memory recall accuracy is affected by retrieval mode.

## Method

### Design

A repeated-measures design was employed in which all participants recorded information about their INMI episodes as they occurred (INMI task) and their VMI when probed by an experimenter (VMI task). Each of the tasks took place over 3 days (72 h) during the course of participants’ daily lives; the two 3-day periods always fell during the course of a time period of no more than 2 weeks. The INMI task was always administered prior to the VMI task, as participants were asked to imagine tunes during the VMI task that they had previously reported as INMI, thus allowing both tasks to utilize the same set of tunes for each participant.

### Participants

Twenty participants were recruited on the basis that they reported experiencing INMI at least a few times per day on average. This sample size was determined via a power analysis conducted at 80 % power using G*Power software (Faul, Erdfelder, Lang, & Buchner, 2007) and effect sizes from the linear mixed effects models reported in Jakubowski et al. (2015). The sample ranged in age from 19 to 34 years (*M* = 23.55, *SD* = 4.49; seven males). The age range was constrained to include only young adults, as previous research has suggested there may be age-related changes in the precision and consistency of musical imagery during older age (Jakubowski, 2015). Scores on the Musical Training dimension of the Goldsmiths Musical Sophistication Index (Gold-MSI; Müllensiefen, Gingras, Musil, & Stewart, 2014) ranged from 9 to 42 (*M* = 25.15, *SD* = 12.02), indicating a wide variety of backgrounds in terms of participants’ formal musical training.^3^

### Materials

A GENEActiv Original accelerometer^4^ was used to collect each participant’s INMI and VMI tempo data. This device resembles a wristwatch and was worn by each participant on his/her dominant hand. The same brand of GENEActiv accelerometers was used in previous research by Jakubowski et al. (2015) to collect INMI tempo data; the present study used the same tapping protocol for participants and analysis procedures for the accelerometer data as Jakubowski et al. (2015).

For the INMI task, participants received a diary with eight questions to be answered each time they experienced an INMI episode (see Appendix 1). They were asked to record the time and date of each INMI episode, the name and performer of the INMI tune, the lyrics or section of the tune stuck in their head, and how recently they had heard the tune played aloud. Participants also rated how the INMI tune had affected their current level of emotional arousal (how alert/relaxed the tune was making them feel) and valence (whether the tune was putting them in a positive/negative mood) on 7-point Likert scales, as well as whether their mood had gotten worse, better, or stayed the same since the tune appeared as INMI. For the VMI task, participants received an analogous diary that they were asked to fill in each time they had been prompted to voluntarily imagine a song (see Appendix 2). The questions used in the VMI task diary were the same as those in the INMI task diary, with some rewording to reflect the different conditions of being asked to voluntarily imagine a song (e.g., “Has your mood changed since the tune came into your head?” became “Has your mood changed since purposefully imagining the tune?” in the VMI task).

In addition, two questionnaires were administered to all participants. From the Gold-MSI, we used three self-report subscales (Musical Training, Active Engagement, and Emotions) to probe relevant musical experiences of the participants. The Musical Training subscale measures several facets of formal musical training, including years of lessons, average daily practice, and music theory training. The Active Engagement subscale measures engagement with music other than formal lessons, such as concert attendance and active listening, and the Emotions subscale measures participants’ subjective ratings of their emotional responsiveness to music. The Involuntary Musical Imagery Scale (IMIS; Floridou, Williamson, Stewart, & Müllensiefen, 2015) was also administered, which measures several facets of the INMI experience, including how frequently a participant experiences INMI, how bothersome (“Negative Valence” factor) or helpful (“Help” factor) they find their INMI experiences, the degree to which their INMI experiences mirror current concerns (“Personal Reflections” factor), and participants’ propensity to move in time to their INMI (“Movement” factor).

### Procedure

The study was advertised via posters and word-of-mouth. Potential participants were asked to express their initial interest in the study via email to the experimenter. Participants were informed that they could participate in the study if: (1) they tended to experience earworms^5^ at least a few times each day and (2) they were between the ages of 18 and 35 years. All participants who met these criteria were asked to indicate two 3-day periods that fell during the course of a 2-week period in which they were most able to complete the tasks of recording information about their earworms and responding to text messages from the experimenter (i.e., they had minimal work or study commitments that would impede on successful completion of these tasks).

Once a participant had indicated his/her two 3-day periods of choice, an initial 20-min meeting was arranged with the experimenter. The experimenter gave an overview of the study requirements and administered two tapping screening/training tasks to the participant. The aims of these tapping tasks were to: (1) ensure that each participant was able to find the beat in a piece music and (2) ensure the participant understood that throughout the INMI and VMI tasks they would be asked to tap to the beat (rather than the rhythm) of imagined music. In the first tapping task, the participant heard one excerpt from each of four pop songs (*Dancing Queen, Bohemian Rhapsody, Hey Jude,* and *Smoke on the Water*), which ranged from approximately 20 to 30 s in length. The participant was asked to tap along to the beat of these songs on the touchpad of a MacBook Air laptop. In the second tapping task, the participant was asked to choose four familiar songs from a list of ten folk/ children’s songs (e.g., *Happy Birthday, Jingle Bells*, etc.). The lyrics to 16 beats of each selected song were provided and the participant was asked to imagine this excerpt in his/her head while tapping along to the beat. The experimenter watched the participant throughout the two tapping tasks and re-explained the task and asked the participant to repeat a trial if: (1) he/she did not maintain a generally steady beat (e.g., switched to double- or half-time in the middle of a trial) or (2) tapped to the rhythm of a song rather than the beat. All participants were able to complete at least 75 % of trials of these two tasks to a level that demonstrated to the experimenter that they understood the concept of finding a beat in both perceived and imagined music. After these tapping tasks, participants were asked to complete the three subscales of the Gold-MSI and the IMIS questionnaire.

Following completion of the questionnaires, the experimenter provided written instructions for the INMI task, including a definition of the term *earworm*. Participants were informed that each time they experienced an earworm over the next 3 days they should tap to the beat of the earworm tune as closely as possible to what they heard in their head with the arm on which they were wearing the accelerometer. They were asked to tap using their full forearm on their leg at least 20–25 times for each earworm episode, followed by a button press on the accelerometer to serve as a marker of the end of each tapping period. They were informed that a button press was not required at the start of a tapping period, which thereby allowed tapping to occur naturally and spontaneously, as soon as participants noticed an earworm. Participants were then asked to trial the tapping procedure and button press with the accelerometer. Participants were asked to wear the accelerometer continuously throughout the 3-day period, excluding periods of sleep and bathing. The experimenter then gave participants the paper diary and explained each question to ensure understanding. Participants were asked to fill in one page of the paper diary as soon as possible following each tapping period.

The participant took away the accelerometer and diary in order to complete the INMI task over the course of the next 3 days. A meeting was scheduled for a convenient time after these 3 days had elapsed in order to return the INMI task materials and begin the VMI task.

The meeting to initiate the VMI task was approximately 5 min in duration. Participants were presented with a different accelerometer of the same make and specifications as the one they had used in the INMI task (to ensure full recording capacity and so the experimenter could begin extracting the participant’s INMI data), written VMI task instructions, and a VMI task diary. Participants were told that during this task they would receive prompts via text messages to their mobile phones, which asked them to voluntarily imagine a section of music that they had previously reported as an earworm. They were asked to tap along to the beat of the imagined music in this task while wearing the accelerometer, using the same procedure they had used in the INMI task (including a button press to mark the end of a tapping period). They were also asked to complete one page of the paper diary as soon as possible following each tapping period.

The text messages that participants received over the next 3 days indicated the name, artist, and section of a tune to imagine, which matched the information provided in their INMI diaries. The text message prompts were set up via an online messaging service called fastsms.^6^ The time at which each text message was sent matched the time the participant had reported experiencing an earworm for that piece of music, in order to control for any potential confounding effects of the time of day at which a song was imagined. The only exception to this procedure was for INMI episodes that had been recorded outside the hours of 9 am to 10 pm. VMI text messages that corresponded to such INMI episodes were moved forward or backward in time to the next closest time that fell within this 9 am to 10 pm time range, so as not to intrude upon participants’ possible sleep schedules; this procedure was implemented for 35 out of 258 episodes (13.6 %). Participants also received a text message each morning during the VMI task informing them of the times to expect text messages,^7^ in an attempt to increase the likelihood that they would make an effort to be available and responsive to the task.

Following completion of the VMI task, participants returned the study materials, were debriefed as to the purpose of the experiment, and received some modest monetary compensation for their time.

### Analysis

#### Tapping data

Tapping data from the accelerometers for both the INMI and VMI tasks were analyzed using an analogous procedure to that reported by Jakubowski et al. (2015). To summarize, each INMI or VMI tapping episode was located manually within the Data Analysis feature of the GENEActiv software using the time and date reported in the diary booklet, with the button press as a marker of the episode endpoint. Each tapping episode was then analyzed using a tap detection algorithm in MATLAB, which identifies taps by performing local maxima detection above a set threshold (see Jakubowski et al., 2015 for full details). The first eight taps were excluded from analysis in each episode, in line with previous tapping literature (e.g., Benoit et al., 2014; Sowiński & Dalla Bella, 2013), and all numerical measurements were calculated based on the remaining taps. If there were fewer than 10 remaining taps after excluding the first eight taps, this was recorded as a missing value, as the tapping period was deemed too short to extract a reliable tempo estimate. The time series of inter-tap intervals (ITI) was calculated as the difference between all successive tap onsets. Artefacts (ITIs of less than 100 ms) and outliers (ITI values greater than three times the interquantile range from the median value of the ITI series) were removed from each ITI series. Finally, an average ITI value, coefficient of variation (CV; a normalized measure of tapping variability defined as the standard deviation of the ITI series divided by the mean ITI), and tempo in beats per minute (bpm) were calculated for each tapping episode.

#### Tempo data from original recordings

For tunes reported as INMI that had a corresponding canonical version (i.e., a definitive, recorded version), the tempo of the section of the tune that was reported as INMI was calculated from the original recording. Recordings of canonical tunes were identified and acquired using music streaming services (Spotify, iTunes, Amazon Music). Audacity sound editing software (2.1.2, 2015, Audacity Team) was used to locate the start and end times of the sections of the tunes reported as INMI, and entered into an Excel spreadsheet where the total section duration was calculated. The number of beats in each section was counted by tapping to the underlying beat and entered into the Excel spreadsheet. Using the total duration and number of beats, the tempo (in bpm) was calculated for each musical excerpt. These calculations were cross-checked by a second researcher to ensure accuracy and consistency.

#### Diary data

Participants’ hand-written diary data were entered into Microsoft Excel for further analysis in Excel and R. Each INMI diary entry was matched with its corresponding VMI diary entry.

## Results

A total of 258 INMI episodes were recorded in the diaries (four to 32 episodes per participant; *M* = 12.9, *SD* = 6.68). INMI was experienced for 175 different pieces of music, comprising a wide range of musical genres (pop, classical, rock, rap, TV jingles, Christmas songs, etc.). Four INMI tunes were reported by more than one participant: *Work* by Rihanna (reported by four participants), *Dancing Queen* by ABBA (three participants)^8^, *One Dance* by Drake (two participants), and *Cheap Thrills* by Sia (two participants). Overall, 36.0 % of reported INMI tunes had been heard aloud (in a recorded or live version) by the participant within the past day, whereas 34.1 % had last been heard over 1 week ago. For VMI tunes, 8.53 % of tunes had been heard aloud in the past day and 22.9 % had last been heard over 1 week ago.

A corresponding, complete VMI diary entry was obtained for 226 of the 258 INMI tunes (87.6 %). Usable tapping data were obtained for both INMI and VMI episodes for 156 of these 226 episodes (69.0 %). This rate of usable data is quite consistent with the rate of usable INMI tapping data reported in Jakubowski et al. (2015) of 82.9 %, as computed across *two* tasks (INMI and VMI) for the present study (82.9 % * 82.9 % = 68.7 %). Tapping data were deemed unusable if no tapping period could be identified during the manual inspection of the accelerometer data, the tapping period was too short (less than 10 taps remained following the exclusion of the first eight taps, artefacts, and outliers), or tapping could not be extracted reliably due to signal glitches or lack of a regular periodicity in the tapping data (e.g., participant may have tapped the rhythm rather that the beat or had trouble finding a steady beat). This was the case for 48 INMI episodes and 26 VMI episodes.

**Fig. 1:**
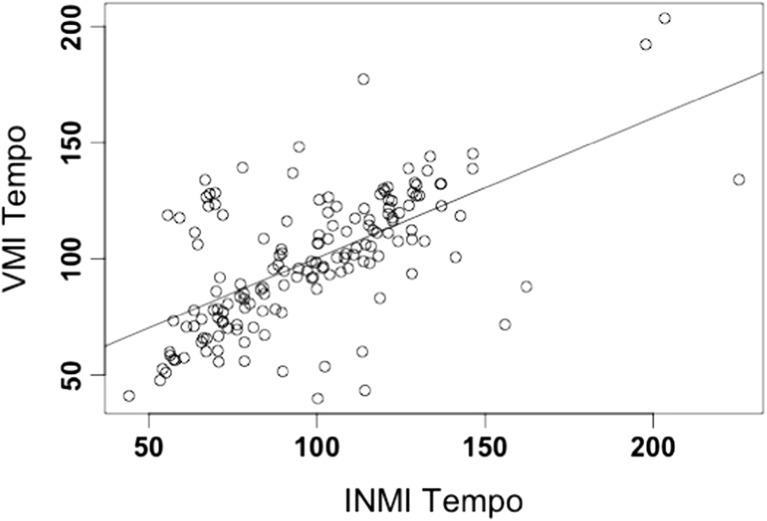
INMI tempo plotted against VMI tempo for the same tune

Two participants did not contribute any usable data to the final INMI/VMI tempo pairings, primarily because their tapping periods were systematically shorter than the required 18 taps. The remaining participants contributed usable data for 25–100 % of their INMI episodes (*M* = 66.5 %, *SD* = 27.3 %), suggesting some individual differences in terms of ability or motivation to perform the tapping task. Since different participants contributed different numbers of INMI episodes, the primary analyses in this paper will be conducted using linear mixed effects models, which use a precision weighted average to model the individual participants as a random factor and thus can account for the multiple and unequal distribution of responses across participants (Janssen, 2012; Raudenbush & Bryk, 2002). The final usable tapping episodes for both INMI and VMI were distributed across a wide tempo range (see Fig. 1) centered around approximately 100 bpm, which aligns with previous evidence indicating human preferred tempo for music is approximately 100–120 bpm, with the mean tempo for large pop music corpora centering around these values (McKinney & Moelants, 2006; Van Noorden & Moelants, 1999).

### Comparing INMI and VMI

#### Descriptive results

Analysis of the 156 episodes for which both INMI and VMI tempo data were obtained revealed a mean INMI tempo of 98.36 bpm (*SD* = 29.94) and a mean VMI tempo of 99.38 bpm (*SD* = 28.92). The mean CV of tapping was 0.063 (*SD* = 0.046) for INMI and 0.059 (*SD* = 0.032) for VMI. Paired-samples t-test indicated there was no significant difference in mean tempo (*t*(155) = 0.497, *p* = .620) or tapping variability (*t*(155) = 0.964, *p* = .337) between the INMI and VMI tasks.

**Fig. 2:**
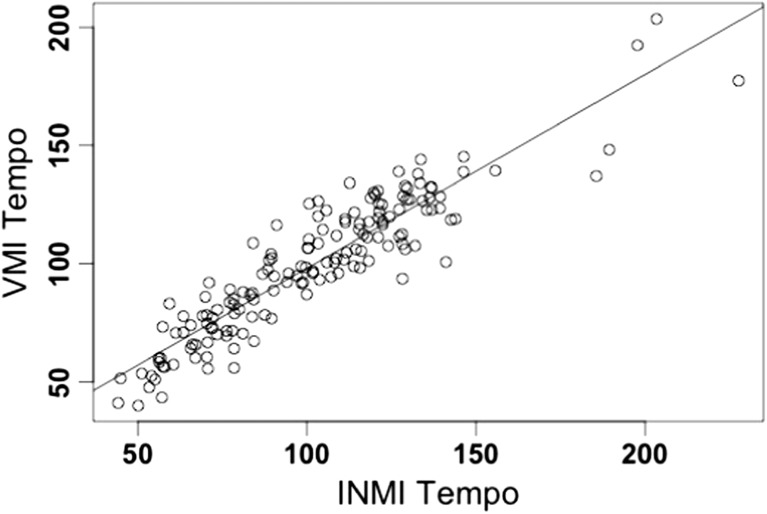
INMI tempo plotted against VMI tempo for the same tune (mapped to the same metrical level)

A positive correlation between INMI and VMI tempo for the same tune was found (*r*(154) = .625, *p* < .001). These results are visualized in Fig. 1. It is evident from these data that some doubling or halving of tapped tempo may have occurred between the two imagery conditions, as the beat of the same musical stimulus can often be perceived at different metrical levels (e.g., McKinney & Moelants, 2006; Parncutt, 1994). If this analysis is recalculated such that the INMI tempo data is transformed to map onto the closest metrical level to the VMI tempo (i.e., INMI tempi is doubled or halved in these cases),^9^ the magnitude of the correlation between INMI and VMI tempo increases even further to *r*(154) = .917 (*p* < .001; see Fig. 2).

#### Tempo accuracy

**Fig. 3:**
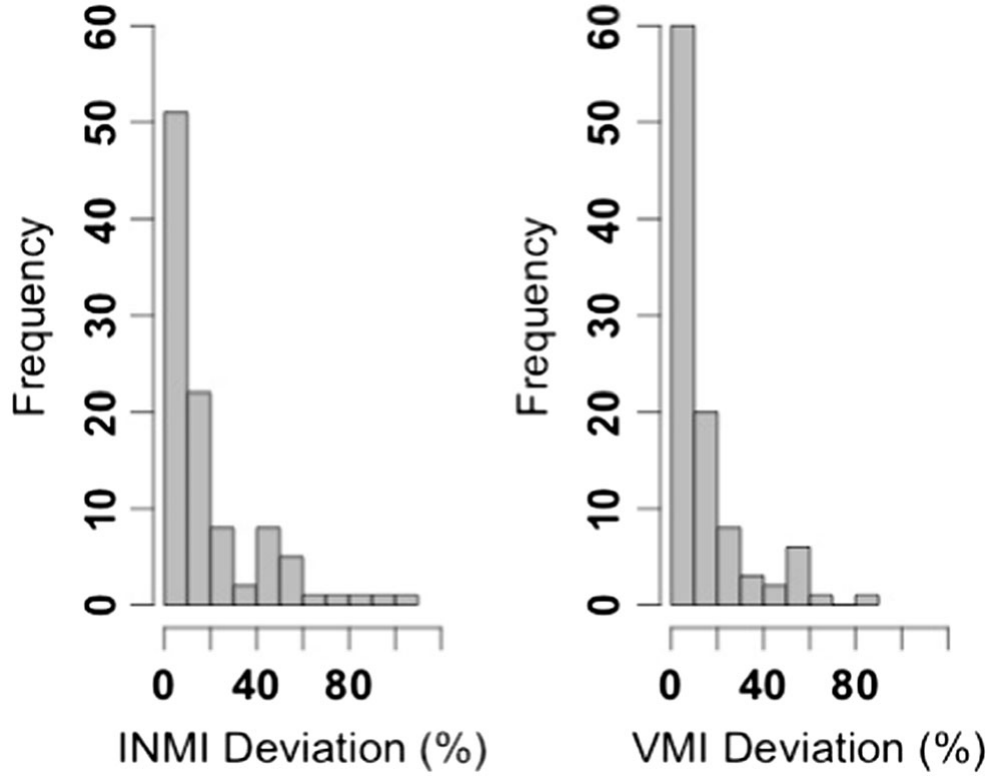
Absolute deviation (as a percentage) of tapped tempo from original, recorded tempo for INMI and VMI

In total, 101 data points for INMI/ VMI pairings of canonical tunes were collected. The tempo of these imagery episodes was compared to the original, recorded tempo of each tune (see Fig. 3). The mean ratio of INMI tempo to the original tempo was 0.96 and the mean ratio of VMI tempo to original tempo was 0.95, indicating that, on average, both types of imagery were tapped slightly slower than the original tempo. The median degree of absolute deviation from the original, recorded tempo was 9.8 % for INMI episodes (*M* = 17.7 %, *SD* = 21.4 %) and 7.7 % for VMI (*M* = 13.8 %, *SD* = 16.5 %). As in the previous analyses, the data in Fig. 3 suggest that some participants tapped at a different metrical level to that at which the researchers designated as the “correct” original tempo of a tune. When the INMI and VMI tempo data are transformed to match the metrical levels designated by the researchers for the original tempo,^10^ the median absolute deviation from the original tempo for INMI is 7.7 % (*M* = 9.0 %, *SD* = 7.4 %) and for VMI is 7.3 % (*M* = 9.2 %, *SD* = 8.2 %).

To test whether imagined tempo accuracy differed as a function of task (INMI/VMI), a linear mixed effects model was fitted with “Task” as a (fixed effect) predictor of absolute deviation from the original tempo and “Participant” as a random effect. Including “Participant” as a random effect allows us to take account of individual differences in response distributions by assuming different baselines for each participant and estimating a separate random intercept for each (Winter, 2013). As accuracy of tempo recall may also be affected by how recently a tune has been heard aloud, self-report ratings of recency of hearing were also included as a predictor in this model. Results indicated no significant effect of task or recency of hearing on deviation from the original tempo and no significant interaction between these two predictors^11^ (see Table 1), suggesting a generally equivalent level of tempo recall accuracy for INMI and VMI that is not influenced by how recently a tune has been heard aloud. If the same analysis is performed using the ratio of tapped to original tempo as the dependent variable (a relative measure that treats over- and under-estimation of tempo separately) rather than the absolute deviation from the original tempo, the same pattern of results holds, with no significant effect of task (*t*(189.4) = -0.156, *p* = .877), recency of hearing (*t*(165.1) = 1.034, *p* = .303), or task by recency interaction (*t*(175.1) = 0.150, *p* = .881).

**Table 1:** Linear mixed effects model with task (INMI/VMI) and recency of hearing as predictors of absolute deviation from the original, recorded tempo

Predictor	Estimate	Standard error	Degrees of freedom	t-value	p-value
Intercept	0.090	0.020	56.5	4.432	< .001
Task	0.029	0.035	94.9	0.831	.408
Recency of hearing	0.003	0.006	134.6	0.576	.566
Task * recency of hearing	-0.010	0.012	102.3	-0.852	.396

*Note:* “Participant” has been included in the model as a random effect

As an exploratory question, we also probed the relationship between imagined tempo recall accuracy and the musical backgrounds of the participants by fitting a second linear mixed effects model with Gold-MSI Musical Training scores and task (INMI/VMI) as predictors of the absolute deviation of tapped tempo from the original tempo and “Participant” as a random effect (see Table 2). No significant effect of musical training was found on tempo recall accuracy and no significant interaction was found between musical training and task, suggesting that less musically trained participants did not demonstrate less accurate imagined tempo representations in either imagery task than trained musicians.

**Table 2:** Linear mixed effects model with task (INMI/VMI) and musical training as predictors of absolute deviation from the original, recorded tempo

Predictor	Estimate	Standard error	Degrees of freedom	t-value	p-value
Intercept	0.107	0.028	12.5	3.862	.002
Task	0.020	0.026	12.2	0.784	.448
Gold-MSI Musical Training	-0.003	0.001	11.1	-0.327	.750
Task * Gold-MSI Musical Training	-0.001	0.001	8.8	-0.816	.436

*Note:* “Participant” has been included in the model as a random effect

#### Emotional responses

**Fig. 4:**
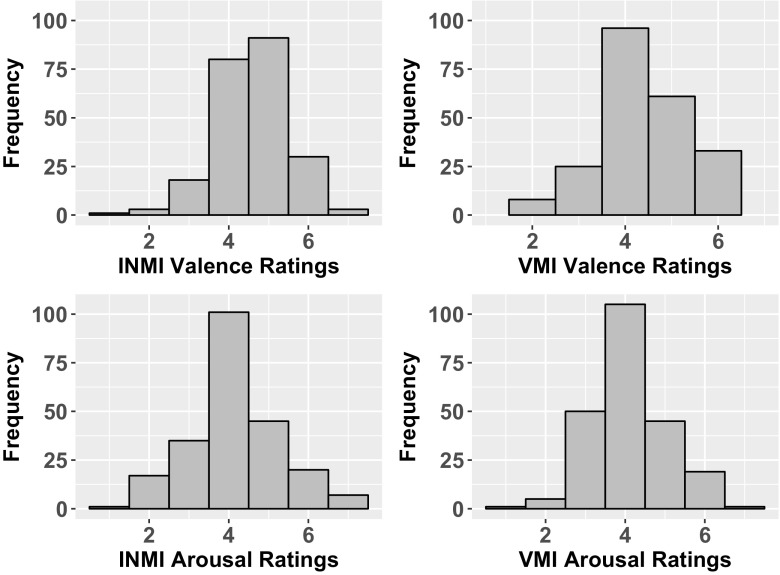
Distributions of valence and arousal ratings for INMI and VMI tunes

Figure 4 displays the distributions of valence and arousal ratings for the 226 INMI/VMI tune pairings for which the diary questions on how the musical imagery was affecting participants’ levels of valence/arousal were answered. On a descriptive level, average ratings of both valence and arousal were higher during INMI (*M*_Valence_ = 4.59, *M*_Arousal_ = 4.15) than VMI (*M*_Valence_ = 4.42, *M*_Arousal_ = 4.10). Pearson correlations were computed for each individual participant to compare instances of the same tune as experienced as INMI versus VMI in terms of valence and arousal ratings. The mean correlation across all participants between valence ratings for the same tune as experienced as INMI versus VMI was .19 and the mean correlation between arousal ratings was .27, suggesting some degree of mood congruence between the two types of imagery. However, the range of these correlations also indicated a large degree of individual differences between participants in terms of whether INMI and VMI for the same tune occurred during similar mood states (range for valence correlations: -.55 to .81, range for arousal correlations: -.42 to .80). To test the effect of task (INMI/VMI) on arousal and valence ratings separately, linear mixed effects models were fitted, with “Participant” as a random effect. These analyses revealed no significant effect of task on arousal ratings (see Table 4), but a marginally significant effect (*p* = .079) of task on valence ratings, such that higher (more positive) valence ratings were found for INMI as compared to VMI (see Table 3).

**Table 3:** Linear mixed effects model with task (INMI/VMI) as a predictor of valence ratings

Predictor	Estimate	Standard error	Degrees of freedom	t-value	p-value
Intercept	4.579	0.083	23.6	55.29	< .001
Task	-0.176	0.098	39.0	-1.80	.079

*Note:* “Participant” has been included in the model as a random effect

**Table 4:** Linear mixed effects model with task (INMI/VMI) as a predictor of arousal ratings

Predictor	Estimate	Standard error	Degrees of freedom	t-value	p-value
Intercept	4.150	0.147	17.9	28.32	< .001
Task	-0.026	0.139	18.5	-0.189	.852

*Note:* “Participant” has been included in the model as a random effect

**Table 5:** Linear mixed effects model with INMI tempo as a predictor of arousal ratings

Predictor	Estimate	Standard error	Degrees of freedom	t-value	p-value
Intercept	3.327	0.342	102.5	9.73	< .001
Tempo	0.008	0.003	149.0	2.70	.008 **

*Note:* “Participant” has been included in the model as a random effect

**= significant at the level of p < .01

**Table 6:** Correlations between average valence/arousal ratings and related questionnaire responses

Questionnaire subscale	INMI valence	INMI arousal	VMI valence	VMI arousal
Gold-MSI Emotions	*r*(18) = .083 (*p* = .729)	*r*(18) = .289 (*p* = .217)	*r*(18) = .087 (*p* = .715)	*r*(18) = .461 (*p* = .041)*
IMIS Negative Valence	*r*(18) = -.494 (*p* = .027)*	*r*(18) = .418 (*p* = .067)	*r*(18) = -.242 (*p* = .304)	*r*(18) = -.153 (*p* = .520)
IMIS Help	*r*(18) = -.096 (*p* = .687)	*r*(18) = -.309 (*p* = .185)	*r*(18) = -.379 (*p* = .100)	*r*(18) = -.158 (*p* = .506)

*= significant at the level of p < .05

A further plausible alternative to assuming that retrieval mode (involuntary/voluntary) would affect emotional responses to all tunes in a similar way is to take account of the musical features of each imagined tune, as emotional responses to perceived music are known to be affected by features such as tempo, mode (major/minor), and dynamics (e.g., Eerola, Friberg & Bresin, 2013; Webster & Weir, 2005). In this particular study, it was possible to examine whether the tempo of the imagined music also affected valence and arousal ratings (for the N=156 INMI/VMI pairings with usable tempo data), and whether this influence was similar across both types of imagery. Initial correlational analyses between imagined tempo and emotion ratings indicated a significant positive correlation between INMI tempo and arousal ratings (*r*(149) = .298, *p* < .001), but no significant correlations between INMI tempo and valence (*r*(150) = -.077, *p* = .346) or VMI tempo and either arousal (*r*(151) = .128, *p* = .116) or valence ratings (*r*(151) = .102, *p* = .210). If the multiple observations per participant are accounted for in linear mixed effects models these same relationships hold: INMI tempo is a significant predictor of arousal (see Table 5), but not valence ratings (*t*(115.1) = -0.931, *p* = .354) and no significant effects of tempo on arousal (*t*(138.0) = 1.49, *p* = .138) or valence ratings (*t*(146.6) = 0.573, *p* = .567) are revealed in the VMI task.

A chi-squared test was conducted to test whether responses to the question “Has your mood changed since the tune came into your head/purposefully imagining the tune?” varied as a function of task (INMI/VMI). No significant association was revealed in this analysis, χ^2^ (2) = 3.80, *p* = .150.

Finally, average valence and arousal ratings from the diary data were correlated with relevant dimensions from the Gold-MSI and IMIS questionnaires in an exploratory examination of individual differences between participants (see Table 6). A significant negative correlation between INMI valence ratings and scores on the Negative Valence factor of the IMIS was found, indicating that participants who self-reported more negative reactions to INMI on the IMIS questionnaire did appraise INMI as a more negative experience during its actual occurrence in the diary study. Also of note is a positive correlation (*r* = .418) between IMIS Negative Valence scores and INMI arousal ratings. Although only marginally significant, this correlation suggests that people who self-report INMI to be more disturbing in general tend to experience greater arousal during INMI, which could relate to feelings of distress or disturbance. No significant associations with general self-reported emotional responsiveness to music (as recorded by the Gold-MSI Emotions subscale) were found, except for a positive relationship between Gold-MSI Emotions scores and VMI arousal ratings. Finally, participants who reported INMI to be a generally helpful experience (IMIS Help factor) did not demonstrate systematic differences in their ratings of INMI valence/arousal than those who found it less helpful.

## Discussion

The present study introduced a new paradigm for comparing involuntary and voluntary retrieval of the same pieces of music by the same participants. Within-subject comparisons of the *same* memory as retrieved both involuntarily and voluntarily have been rare in the memory literature; such comparisons are particularly feasible within a music research context due to the ubiquity of INMI and the ease with which specific songs can be brought to mind deliberately as VMI. In our study, comparisons between INMI and VMI were made specifically in terms of accuracy of musical tempo recall and emotional responses. It was found that INMI and VMI for the same tune were recalled with a similar degree of temporal accuracy in comparison to its original, recorded version. Some evidence for heightened emotional responses to INMI was found, including that musical aspects (tempo) of the imagery were more systematically related to emotional responses in the INMI than the VMI task.

A strong correlation was found between the tempo of INMI and VMI for the same tune. This supports the proposition that involuntary memories rely on the same memory system as voluntary memories and differ only in retrieval processes (Berntsen, 2010) and extends this account into the domain of involuntary musical memories. The present study was also the first to our knowledge to compare accuracy of involuntary versus voluntary musical memories. No significant difference was found in accuracy of tempo recall between INMI and VMI. This suggests that spontaneous occurrences of music in the mind are no less precise (at least in terms of tempo recall) than instances in which participants are instructed to deliberately and strategically call a tune to mind. These results parallel those of Mace et al. (2011), who found subjective ratings of confidence in the details of memories did not differ between IAMs and VAMs, and extends this finding to the comparison of the exact same memory as retrieved both involuntarily versus voluntarily. The autobiographical memory literature also suggests that IAMs tend to be more specific and accompanied by greater reliving than VAMs. Although this may potentially be the case for INMI as compared to VMI, it does not appear that this difference is manifested as a difference in recall *accuracy*, at least when comparing INMI and VMI for the same tune. Future studies might also examine vividness ratings as a potential point of divergence between the two types of musical imagery, as well as whether naturally occurring instances of VMI (rather than those prompted by an experimenter to match the tune recalled in the INMI task) differ systematically from INMI.

On average, INMI and VMI were both recalled with high accuracy in comparison to the original, recorded tempo for canonical tunes (INMI median deviation from original tempo = 7.7 %; VMI median deviation = 7.3 %). The results of the INMI task closely replicate the findings of Jakubowski et al. (2015), who reported a median deviation of INMI tempo from the original tempo of 7.9 %. In another previous study on accuracy of tempo recall, Levitin and Cook (1996) asked participants to sing two familiar, self-selected pop songs and found that 72 % of trials were sung within 8 % of the original, recorded tempo. Tempo recall in the present study was slightly less precise (50 % of INMI episodes and 56 % of VMI episodes in the present study were within 8 % of the original tempo), although this could potentially be attributed to the heightened familiarity and perceptual feedback afforded by Levitin and Cook’s task. In addition, both types of imagery in the present study tended to be imagined slower than the original, recorded tempo. This replicates the results of Jakubowski et al. (2015) in regard to INMI tempo. One potential explanation for this result could be the motor constraints of the tapping task; future research could explore whether non-motor methods for measuring tempo elicit a similar result.

Accuracy of tempo recall was not affected by recency of hearing a tune aloud in either imagery task. Jakubowski et al. (2015) also found no effect of recency of hearing on INMI tempo accuracy; our study extends this result to voluntarily retrieved imagery. It may be that INMI tends to be experienced for music that is highly familiar or “overlearned,” thus long-term memory representations are relatively strong and stable and recent hearing does not increase the precision of tempo recall. Such well-learned pieces would also likely be recalled with high precision in a VMI task without requiring recent hearing to reinforce memory representations. Future work could also consider the effects of recency of *imagining* a tune (e.g., whether recent INMI could affect the mental representation of VMI for the same music), although given the various parallels between music listening and musical imagery (e.g., Zatorre & Halpern, 2005), it is not anticipated that recent imagining will have different effects to recent hearing. In addition, no relationship between formal musical training and accuracy of tempo recall was found in either imagery task, indicating that this high precision of imagined music recall is not affected by domain-specific expertise, although future research should investigate this relationship further in a larger sample with a more diverse range of musical backgrounds.

In terms of emotion ratings, INMI and VMI of the same tune elicited similar ratings of valence and arousal for some but not all participants. A marginal effect of task on valence ratings indicated that more positive emotional responses were elicited by INMI than VMI. This finding contradicts the common stereotype of “earworms” as a necessarily annoying disturbance, but aligns with previous findings that most INMI episodes are rated as pleasant or neutral in valence (Beaman & Williams, 2010; Halpern & Bartlett, 2011). In addition, some previous studies have found that IAMs were reported as more emotionally positive than VAMs (Berntsen, 1998), although this finding has not always replicated across different samples and tasks (Berntsen & Hall, 2004). A significant effect of INMI tempo was found on arousal ratings, which replicates Jakubowski et al. (2015), who also found that INMI tempo was significantly related to arousal but not valence. Conversely, no effect of VMI tempo was found on arousal ratings, suggesting that the musical features of INMI may have a more emotional impact than the same features of a tune when recalled as VMI. This is in line with the proposition that the unprepared nature of involuntary memories can elicit heightened emotional responses in comparison to deliberately retrieved memories (Berntsen, 2010).

Finally, some novel correlational results were revealed in relation to self-reported appraisal of the INMI experience and actual emotion ratings during an INMI episode. A significant correlation between scores on the IMIS Negative Valence factor and valence ratings of INMI during the diary study provides further validation that this retrospective, self-report questionnaire is able to capture individual differences in INMI appraisal. It was also found that participants with higher scores on the IMIS Negative Valence factor gave higher ratings of INMI-induced arousal. Although directionality of this relationship cannot be established from correlational data, one possibility could be that individuals who find INMI particularly unpleasant are those whose arousal levels are disproportionately affected by the INMI episode or those for whom there is a lack of contextual fit, such that an INMI episode induces high levels of arousal at inopportune moments (e.g., when one is attempting to fall asleep). Anecdotal reports from individuals who are particularly disturbed by INMI would support this suggestion. Future research should continue to explore the relationship between results on self-report questionnaires such as the IMIS and naturalistic, real-time responses (using diary or Experience Sampling Methods) within the same participant in an attempt to further understand individual differences in responses to INMI.

In conclusion, the present work introduced a novel paradigm for studying memories in which the only factor that substantially varied between the two tasks was the retrieval mode (involuntary vs. voluntary). This approach revealed new evidence indicating that musical memories are recalled with a similar degree of accuracy regardless of retrieval mode, with some differences in emotional responses to the same memory as recalled involuntarily versus voluntarily. Such research offers a valuable opportunity to extend the comparison of involuntary and voluntary retrieval to the domain of involuntary semantic memories and to isolate the effect of retrieval mode when the memory itself is held constant.

### Author Note

This research was funded by the Society for Education, Music and Psychology Research (SEMPRE), through a Reg and Molly Buck award to author KJ.

## Footnotes

1 See Rubin and Berntsen (2009) for an exception, in which the frequency of IAMs and VAMs related to the same event was examined. However, this approach is still not entirely analogous to the present study, in that participants were asked to estimate the frequency of IAMs and VAMs related to a certain event (e.g., their confirmation day), but may still have experienced a variety of different memories related to that event (e.g., memories of getting ready at the start of the day, memories of the confirmation service itself, memories of the after-party, etc.).

2 An example of a canonical tune reported as INMI in the present study is *Dancing Queen* by ABBA; an example of a non-canonical tune that was reported is *Jingle Bells.*

3 The possible range of scores on the Musical Training dimension of the Gold-MSI is 7 to 49, with 49 indicating the highest level of musical training.

4 http://www.geneactiv.org/

5 The term *earworm* was used in all communications to participants, as it was deemed a more familiar and colloquial term than INMI. All participants were provided the following definition of an earworm: “An earworm is a short section of music that comes into your mind without effort (it is involuntary; without any intention to retrieve or recall the music) and then repeats by itself (immediately repeated at least once, on a loop, without you consciously trying to replay the music).”

6 http://www.fastsms.co.uk/

7 Only a list of times was given in this text message; no information on the tunes to be imagined was given at this point.

8 It should be noted that this tune was used as a stimulus in the tapping screening/training task in the laboratory prior to the start of the INMI task, thus participants may have been primed by recent exposure to experience this tune as INMI.

9 Specifically, if the doubled INMI tempo value was closer to the VMI tapped tempo than the raw INMI tempo value, then the INMI tempo value was doubled. If the halved INMI tempo value was closer to the VMI tapped tempo than the raw INMI tempo value, then the INMI tempo value was halved. For example, in one case an INMI value of 55.635 bpm was doubled to 111.27 bpm when the corresponding VMI tempo value was 118.81 bpm. This resulted in changes to 24 INMI tempo values (15 % of the data).

10 This process is described in footnote 9. This transformation resulted in doubling or halving of 20 INMI and 12 VMI tempo values, comprising 16% of the 202 total imagery episodes.

11 This analysis was performed on the imagery tempo data that was transformed to the same metrical level as the original, recorded tempo data. All predictors remain non-significant when the same analysis is performed on the raw data (*p*s > .05).
